# Exploring Dangerous Connections between *Klebsiella pneumoniae* Biofilms and Healthcare-Associated Infections

**DOI:** 10.3390/pathogens3030720

**Published:** 2014-08-19

**Authors:** Maria Bandeira, Patricia Almeida Carvalho, Aida Duarte, Luisa Jordao

**Affiliations:** 1 Departamento de Engenharia Química, Instituto Superior Técnico, Universidade de Lisboa, Av Rovisco Pais, 1049-001 Lisboa, Portugal; E-Mails: maria.bandeira@tecnico.ulisboa.pt (M.B.); pac@ist.utl.pt (P.A.C.); 2 Departamento de Microbiologia e Imunologia; iMed.UL, Faculdade de Farmácia, Universidade de Lisboa, Av Prof Gama Pinto, 1649-003 Lisboa, Portugal; E-Mail: aduarte@ff.ul.pt; 3 Departamento de Doenças Infeciosas; Instituto Nacional de Saúde Dr Ricardo Jorge, Av Padre Cruz, 1649-016 Lisboa, Portugal

**Keywords:** biofilm, healthcare-associated infections (HAI), antibiotic resistance, scanning electron microscopy (SEM)

## Abstract

Healthcare-associated infections (HAI) are a huge public health concern, particularly when the etiological agents are multidrug resistant. The ability of bacteria to develop biofilm is a helpful skill, both to persist within hospital units and to increase antibiotic resistance. Although the links between antibiotic resistance, biofilms assembly and HAI are consensual, little is known about biofilms. Here, electron microscopy was adopted as a tool to investigate biofilm structures associated with increased antibiotic resistance. The *K. pneumoniae* strains investigated are able to assemble biofilms, albeit with different kinetics. The biofilm structure and the relative area fractions of bacteria and extracellular matrix depend on the particular strain, as well as the minimal inhibitory concentration (MIC) for the antibiotics. Increased values were found for bacteria organized in biofilms when compared to the respective planktonic forms, except for isolates Kp45 and Kp2948, the MIC values for which remained unchanged for fosfomycin. Altogether, these results showed that the emergence of antimicrobial resistance among bacteria responsible for HAI is a multifactorial phenomenon dependent on antibiotics and on bacteria/biofilm features.

## 1. Introduction

In recent years, healthcare-associated infections (HAI), defined as infections occurring after exposure to healthcare, but not always as a consequence of this exposure, have been reported as a major public health problem. In 2012, the European Centre for Disease Control (ECDC) has estimated that at least 2.6 million cases of HAI occur annually in long-term care facilities. This number adds to the previously estimated 4.1 million cases in acute-care hospitals, which result in 37,000 annual deaths in Europe [1].

The incidence of HAI varies with body site and is determined to a large extent by the underlying disease condition of patients (e.g., immunosuppression) in addition to their exposure to high-risk medical interventions, such as surgery or invasive diagnostic procedures. Nevertheless, the main infection sites are the urinary and lower respiratory tracts, surgical sites and the bloodstream. Treatment of HAI is challenging due to the prevalence of antibiotic-resistant bacteria [2], and their ability to assemble biofilms can worsen the situation turning HAI refractory to antibiotherapy. Biofilm is defined as a thin layer of microorganisms and secreted polymers adhering to a biotic or abiotic surface, presenting an internal organization that evolves over time. Biofilms tend to increase bacterial resistance to host defense mechanisms, antibiotics, sterilization procedures (other than autoclaving) and the persistence in water distribution systems and in generic surfaces [3].

Biofilm-associated microorganisms have been related with several human diseases and are known to colonize a wide variety of medical devices [4]. *Klebsiella pneumoniae* is a worldwide leading cause of hospital-acquired urinary tract infections, as well as pneumonia, being responsible for many cases of pyogenic liver abscess or endophthalmitis contracted in community patients [5]. The incidence of *K. pneumoniae* infections increased in hospitals and, according to the latest data from ECDC, was included among the six ESKAPE bacteria responsible for two-thirds of all HAIs. The ESKAPE pathogens are multi-drug resistant strains of *Enterococcus faecium*, *Staphylococcus aureus*, *Klebsiella* species, *Acinetobacter baumannii*, *Pseudomonas aeruginosa* and *Enterobacter* species [6]. In spite of their close relation, one notable difference between *K. pneumoniae* and the other members of HAIs is the extremely thick, hypermucoviscous, extracellular polysaccharide capsule. Virulent strains have been predominantly associated with the K:1 and K:2 capsular serotypes. The capsule is believed to be a major virulence determinant by protecting *K. pneumoniae* against phagocytosis and destruction by antimicrobial peptides [7,8]. Other virulence factors have provided new insights into the pathogenic strategies of *K. pneumoniae*, such as fimbriae type 1 and type 3, which mediate attachment to the host mucosal surfaces and inert surfaces [9,10]. In addition, the incidence of isolation of antibiotic-resistant *K. pneumoniae* has increased in recent years, thus complicating the therapy of HAIs and community infections. Research into new antibiotics, phage therapy and vaccines has been some of the features of the past decade. There is now a pressing need for new therapeutic approaches, given the increased number of multi-resistant bacteria (MRB). Here, we focus on the role played by biofilm organization on antibiotic resistance. A deeper understanding of this topic is the first step towards the development of more effective, either preventive of curative, approaches to minimize the impact of HAI.

## 2. Results and Discussion

### 2.1. Bacteria Characterization

Ten *K. pneumoniae* MRB were collected at Lisboa hospitals, between 1980 and 2011 (Table 1). Kp45 and Kp26 strains were isolated, from a nurse neck swab and from a newborn rectal swab, respectively, during a colonization study at a neonatology ward. These isolates showed the same capsular type, K:2. The non-capsulated Kp703 strain was isolated from the urine of a burn patient, and its bacterial surface carbohydrates (O antigen) were typed as O:1. From the remaining isolates, only Kp2948 showed capsular type K:2.

**Table 1:** Characteristics of *Klebsiella pneumoniae* multidrug-resistant isolates producing β-lactamases.

Strain	Source	Year	Serologic Group	Fimbriae		β-lactamases
Kp45	Neck swab	1980	K:2	fimH	mrkD	TEM-1
Kp26	Rectal swab		K:2	n.a.	n.a.	TEM-1
Kp703	Urine		O:1	n.a.	mrkD	TEM-1
Kp3921		2010	n.a.	fimH	mrkD	CTX-M-15
Kp2948	Wound		K:2	fimH	mrkD	KPC-3; TEM-1
Kp3421	Urine	2011	n.a.	fimH	mrkD	CTX-M-15
Kp3407			n.a.	fimH	mrkD	KPC-3
Kp3466			n.a.	n.a.	n.a.	TEM-163
Kp3385			n.a.	fimH	mrkD	KPC-3

n.a.: no amplification.

Various fimbrial adhesins have been shown to play a role in biofilm formation [11]. Most *K. pneumoniae* isolates express two types of fimbrial adhesins: type 1 and type 3. In this study, all strains, except Kp26 and Kp3466, were amplified for fimH and mrkD gene subunits of the type 1 and type 3 fimbrial adhesins, respectively. It should be emphasized that the fimH gene was not found in the non-capsulated *K. pneumoniae* Kp703 strain (Table 1).

The main difference between *K. pneumoniae* strains isolated in 1980 and twenty years later is the antimicrobial resistance; isolates from 1980 were susceptible to all cephalosporins and produced a broad spectrum β-lactamase TEM-1, while isolates from 2010 to 2011 have evolved to encode extended-spectrum β-lactamases CTX-M-15 and TEM-163, which confer resistance to extended-spectrum cephalosporins, with the hallmark being the resistance to ceftazidime and cefotaxime. The Kp2948 and Kp3385 isolates exhibit increased resistance to carbapenem antibiotics, producing KPC-3 carbapenemase. All strains have acquired resistance mechanisms to other classes of antibiotics and are considered MRB.

### 2.2. Biofilm Assembly

**Figure 1:**
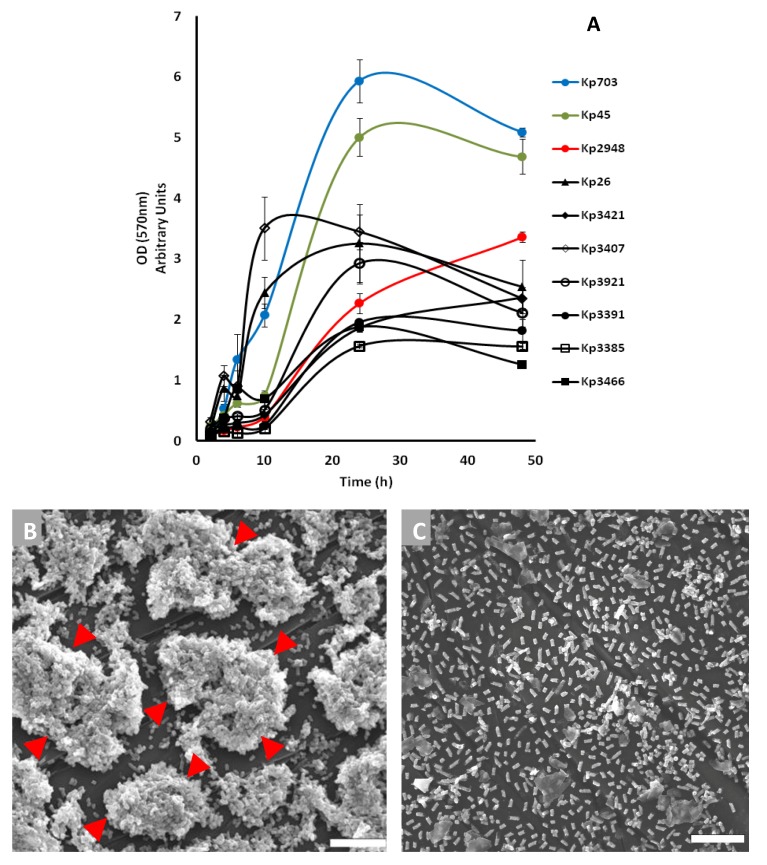
The kinetics of biofilm assembly by 10 strains of *K. pneumoniae* was followed over 48 h using a spectrophotometric assay (**A**); in parallel, scanning electron microscopy (**B**,**C**) was used to illustrate biofilm assembly. Representative micrographs of 12 h-old biofilms of kp703 (**B**) and Kp2948 (**C**) show the different ability to assemble biofilms exhibited by these strains. The presence of organized bacterial structures is highlighted by arrowheads (**B**). Scale bar: 10 μm.

The bacterial ability to assemble biofilms on cell culture plates was evaluated. All strains were able to assemble biofilms, although following different kinetics (Figure 1A). The strains were divided into two main groups according to biofilm evolution. Group 1 includes the strains for which the three major biofilm assembly stages could be clearly identified before 48 h of culture, namely adhesion, maturation and dispersion: Kp703, Kp45, Kp3921, Kp3466, Kp3391, Kp3407 and Kp26. Group 2 comprises Kp2948, Kp3421 and Kp3385, which did not reach the dispersion stage at 48 h.

The most efficient biofilm assemblers were selected from each group for detailed study: Kp703 and Kp45 from Group 1 and Kp2948 from Group 2. *K. pneumoniae* strains Kp703 and Kp45 followed similar kinetics with comparable biomass increase (Figure 1A), although these strains differ in capsule expression; Kp703 is non-capsulated (O:1), while Kp45 is capsulated (K:2) (Table 1).

The Kp2948 strain selected from Group 2 shared several features with Kp45, such as the presence of a K:2 capsule and type 1 (fimH) and type 3 (mrkD) fimbriae; nonetheless Kp2948 exhibited distinct biofilm assembly kinetics (Table 1). Both capsulated strains exhibited longer adaptation phases, supporting the previous findings of the downregulation of type 1 fimbriae [9]. On the other hand, the non-capsulated Kp703 strain had a shorter adaptation phase with a faster increase of biomass. The absence of type 1 fimbriae and the presence of type 3 fimbriae may account for the observed difference in kinetics. Type 3 fimbriae are known to be important, both for initial cell-surface attachment and for the cell-cell adherence mediation in the biofilm [9].

### 2.3. Scanning Electron Microscopy

Scanning electron microscopy (SEM) highlighted the differences between the biofilms assembled by the selected bacterial strains. Micrographs of 12 h-old biofilms are shown in Figure 1. The Kp703 biofilm is fully mature with clear bacterial complexes highlighted by arrowheads in Figure 1B. The Kp2948 biofilm showed few bacteria attached to the surface (Figure 1C), supporting the assumption that this isolate is in a different stage of biofilm assembly (Figure 1A).

**Figure 2:**
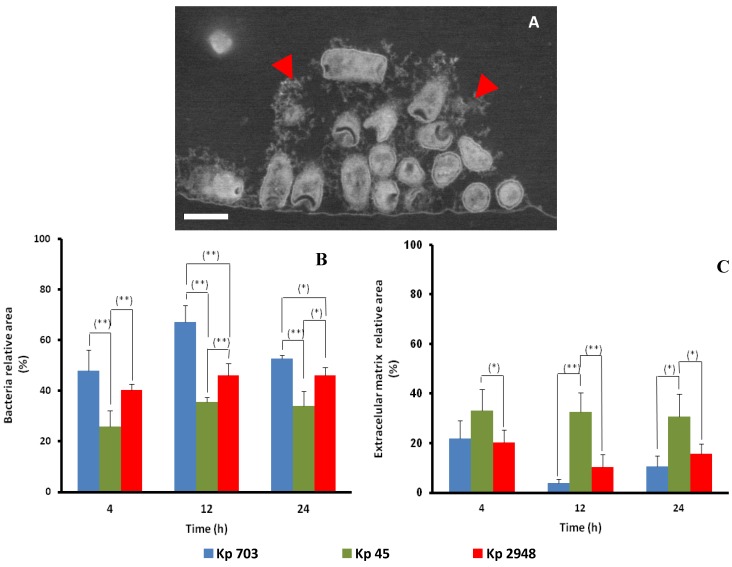
The biofilms assembled by three *K. pneumoniae* strains (Kp45, Kp703 and Kp2948) were characterized using SEM in backscattered electron mode. The existence of extracellular matrix (arrow heads) surrounding bacteria from early stages is highlighted by arrow heads in a 4 h-old Kp703 micrograph (**A**). The relative areas occupied by bacteria (**B**) and extracellular matrix (**C**) during the different phases of biofilm assembly were assessed. The differences were considered significant for *p* < 0.05 (* *p* < 0.05; ** *p* < 0.01). Scale bar: 1 μm.

In order to further characterize the internal organization, biofilm cross-sections were observed in backscattered electron mode after staining with heavy metals. This experimental approach has advantages and disadvantages when compared with transmission electron microscopy (TEM) observations; on the one hand, it is less time consuming and allows larger fields of view; on the other hand, the resolution attained is lower [12]. The method was used to assess the evolution of the relative area fractions of bacteria and extracellular matrix (EPS) as inferred from the cross-sectional areas. A representative micrograph of a 4 h-old Kp703 biofilm is shown in Figure 2A, where EPS is indicated by arrowheads. The results obtained from the analysis of biofilms at different maturation stages are shown in Figure 2B,C, where it is evident that the relative area fraction of bacteria depends consistently on the strain: Kp703 biofilms have higher amounts of bacteria (Figure 2B) than Kp45 biofilms (Figure 2C). In good agreement with the biofilm assay (Figure 1A) and the SEM micrographs shown in Figure 1B, the Kp703 strain was associated with the highest biomass at all stages of biofilm assembly (Figure 2B). At 4, 12 and 24 h, the relative area occupied by Kp45 biofilms is statistically smaller (*p* < 0.010) than in both Kp703 and Kp2948 biofilms. In fact, the relative bacterial areas for the most and least efficient biofilm assembler (Kp703 and Kp2948, respectively) only differ at later stages, when the relative area occupied by Kp703 becomes bigger than the one taken by Kp2948 (*p* < 0.039). These data suggest that Kp703 and Kp2948 assembled similar biofilms, although following different kinetics. The intermediate biofilm assembler (Kp45) registered the lowest bacterial areas for all culture times. Altogether, these results showed that Kp45 biofilm is unique although assembled with a kinetic similar to Kp703 biofilm (Figure 1A).

The area fraction of EPS (Figure 2C), a key player in biofilm assembly and persistence, was also determined from cross-sectional observations. Kp45 excreted significantly more EPS when compared to Kp2948 for all culture times (*p* < 0.048). The EPS relative area fraction in the Kp703 biofilm was always lower than in the Kp45 biofilm; nevertheless, the difference was significant only at 12 and 24 h. No differences between the EPS relative area fractions of Kp703 and Kp2948 were detected. This fact supports the previous assumption that these two strains form similar biofilms, although following different kinetics. The EPS amount was not uniform in each biofilm and may be altered in space and time, proving that different bacteria produce it in different amounts [3]. The EPS content within biofilm varies over time, with the highest amount at the attachment and dispersion phases. Studies have shown that EPS plays an important role in attachment and dispersion phases, binding cells to a surface [3,13]. It should be remembered that the dispersion stage occurs due to a local nutrient depletion, triggering bacteria to colonize other areas from the surface [3]. Extracellular matrix contributes to the increased virulence of microorganisms, blocking mass transport of antibiotics through the biofilm [3] and hampering the elimination of infection by the immune system and antimicrobials.

### 2.4. Antimicrobial Activity of Planktonic and Biofilm-Embedded K. pneumoniae Strains

Biofilms can physically protect bacteria from antimicrobial exposure when compared with planktonic forms, it being important to develop accurate methodologies to determine the MIC for *K. pneumoniae* strains embedded in the respective biofilms.

The MIC values found were different according to antibiotics and bacteria. In general, an increase of MICs for bacteria in biofilm form was verified (Table 2). The increase in MIC values for amoxicillin ranged from five- to 10-fold. The source of β-lactamases in biofilms has been attributed to bacteria lysis promoted by antibiotics. These enzymes once secreted maintain their activity within the biofilm matrix, hydrolyzing β-lactam antibiotics before these drugs reach the bacterial cells [14]. Antibiotics penetration within biofilms could be hampered by other factors, such as the presence of negatively-charged surfaces, particularly for large polar molecules that are positively charged, e.g., aminoglycosides [15]. In addition, limited oxygen and metabolic rates are probably important factors contributing to increased bacterial aminoglycosides tolerance. Altogether, these factors could account for the variation of two-, eight- and 250-fold in MIC values for gentamicin for *K. pneumoniae* isolate Kp2948, Kp45 and Kp703 biofilms when compared to the planktonic forms, respectively. In contrast to amoxicillin and gentamicin, fosfomycin is known to penetrate through biofilm layers [16]. In the present study, only Kp703 increased the MIC value 1000-fold for fosfomycin between planktonic and biofilm forms. The other *K. pneumoniae* strains retained unchanged MIC values for this antibiotic. This apparently unexpected result could be explained by the presence/absence of a capsule. Both capsulated strains (Kp45 and Kp2948) expressing capsular type K:2 showed unchanged MIC values of 0.781 µg/mL, whereas the non-capsulated Kp703 strain registered the most significant increase. The isolate Kp2948 producing carbapenemase KPC-3 is resistant *in vitro* to all β-lactams, including β-lactam/β-lactamase inhibitor combinations, quinolones and aminoglycosides [17]. The therapeutic options are often limited to tigecycline and colistin, although this strain has moderate susceptibility to these antibiotics and shows susceptibility to fosfomycin (data not shown). In summary, fosfomycin demonstrated *in vitro* activity against the KPC-producing *K. pneumoniae* isolate in planktonic and biofilm forms. Fosfomycin is an “old” antimicrobial that rapidly penetrates tissues [18] and could represent a possible alternative to tigecycline and colistin, minimizing the impact of HAI.

Factors affecting biofilm formation were not evaluated in this study. Several genes involved in biofilm formation by *K. pneumoniae* were described, which account for the production of exopolysaccharides, lipopolysaccharides and capsule, as well as the cell density-dependent regulation by quorum sensing [19].

**Table 2:** Comparison of minimal inhibitory concentration (MIC) obtained for planktonic and biofilm-organized *K. pneumoniae*.

**A: MIC (Planktonic)**				
	**Drug**	**Amoxicillin** (**μg/mL**)	**Fosfomycin** (**μg/mL**)	**Gentamicin** (**μg/mL**)
**Strain**				
Kp45		250	0.781	3.05
Kp703		250	<0.488	0.760
Kp2948		>500	0.781	1.52
**B: MIC (Biofilm)**				
	**Drug**	**Amoxicillin** (**μg/mL**)	**Fosfomycin** (**μg/mL**)	**Gentamicin** (**μg/mL**)
**Strain**				
Kp45		>2,500	0.781	24.4
Kp703		>2,500	500	195
Kp2948		2,500	0.781	3.05

## 3. Experimental Section

### 3.1. Bacterial Strains

Ten *Klebsiella pneumoniae* multiresistant strains were isolated from biological products and are part of the FFUL (Faculty of Pharmacy ULisboa, Lisboa, Portugal) collection. All strains were from healthcare-associated infections.

### 3.2. Capsular Type

Three *K. pneumoniae* isolates identified in 1980 (Kp 45, Kp26, Kp703) were typed at the World Health Organization International *Escherichia* and *Klebsiella* Centre, Copenhagen, Denmark. The detection of capsular type K:1 and K:2 for remaining isolates were performed by PCR using specific primers for rmpA [20] and K2a [21] genes, respectively.

### 3.3. Biofilm Assay

The assay was performed in triplicate using 96-well flat-bottomed cell culture plates (Nunc, New York, NY, USA) as described previously with small modifications [22]. Briefly, *K. pneumoniae* suspensions at a final concentration of 10^7^ CFU/mL were prepared in 0.9% sodium chloride from overnight cultures in Mueller–Hinton (MH) agar and ten-fold diluted in MH broth (Oxoid, Basingstoke, UK). Two-hundred microliters were distributed to each well, MH broth being used as the negative control. The plates were incubated at 37 °C to allow biofilm formation for different time periods. Then, the content of each well was removed, and each well was vigorously washed three times with sterile distilled water to remove non-adherent bacteria. The attached bacteria were stained for 15 min with 100 μL of violet crystal at room temperature, washed with distilled water three times to remove excess dye and allowed to dry at room temperature. The violet crystal was dissolved in 100 μL of 95% ethanol (Merck, Damstadt, Germany), and the optical density at 570 nm was read using a (SpectraMax 340PC; Molecular Devices, Sunnyvale, CA, USA).

### 3.4. Scanning Electron Microscopy (SEM)

For SEM analysis, biofilms were allowed to form on six-well cell culture plates (Nunc) for 12 h at 37 °C. The biofilm was fixed with 2.5% glutaraldehyde (EMS, Hatfield, PA, USA), 4% paraformaldehyde (Sigma, St Louis, MO, USA) and 0.05% ruthenium red (Sigma) in 0.1 M cacodylate buffer, pH 7.2, overnight, at 4 °C. This was followed by post-fixation in the dark with 1% osmium tetroxide (EMS), 0.05% ruthenium red and then washed twice with cacodylate and water [23], dehydrated, transferred to glass slides (bioMérieux, Marcy l’Etoile, France) and allowed to dry at room temperature. For backscattered electron analysis, samples were further embedded in Epon812 epoxy resin (EMS) and allowed to polymerize at 65 °C for 3 days. Once polymerized, the blocks were trimmed and sectioned using an ultramicrotome (Leica, Solms, Germany). Thin sections were transferred to coverslips coated with 0.5% (m/v) gelatine (Sigma) and 0.05% (m/v) chromium potassium sulfate dodecahydrate (Panreac, St Loius, MO, USA) and allowed to dry at room temperature. The sections were contrasted with saturated uranyl acetate in water, for 30 min, followed by Reynolds lead citrate for 3 min.

Samples were mounted on the sample holder with carbon tape, sputter-coated with carbon (20 nm) using a Sputter Coater QISOT ES (Quorum Technologies, Laughton, UK) and analyzed under an electron microscope, JSM-7100F (JEOL, Tokyo, Japan).

### 3.5. Antibiotic Susceptibility Test

The antimicrobial activity was evaluated by the micro-dilution method according to Clinical and Laboratory Standards Institute guidelines, using amoxicillin (Bio-Rad, Hercules, CA, USA), fosfomycin (Bio-Rad) and gentamicin (Gibco, New York, NY, USA). Briefly, antibiotics were diluted in MH broth to produce a two-fold dilution in the concentration ranges from 10,000 to 0.0048 µg/mL for amoxicillin, 500 to 0.244 µg/mL for fosfomycin and 12,500 to 0.191 µg/mL for gentamicin. A positive control containing a suspension of bacteria in MH broth without antibiotics was performed in parallel. The minimum inhibitory concentration (MIC) was defined as the lowest concentration of antibiotic resulting in the absence of turbidity after over-night incubation at 37 °C.

The MIC for biofilm was performed using the same antibiotics and concentrations. Briefly, a biofilm was allowed to form as described in Section 3.2 during 12 h. After removing the non-adherent bacteria, the antibiotic solutions were added, and the plate was sonicated in a water table sonicator for 5 min and incubated over-night at 37 °C.

### 3.6. Statistical Analysis

The results of at least three independent experiments were expressed as the means ± standard deviation (SD). SEM micrographs were analyzed using Image J software with the statistical significance assessed by the Student *t*-test (two-tailed). A *p*-value of <0.05 (*) and <0.01 (**) were considered statistically significant.

## 4. Conclusions

This study supports the hypothesis that biofilms formed on medical devices can promote the onset and enhance the spread of HAIs. The biofilm assembly process is bacterial type specific. Biofilm-forming bacteria are generally more resistant to antibiotics; however, fosfomycin demonstrated *in vitro* activity against KPC-producing *K. pneumoniae* isolate, both in planktonic and biofilm forms. The emergence of antimicrobial resistance among bacteria responsible for HAIs is a multifactorial phenomenon dependent on antibiotics and bacteria/biofilm features. Therefore, elucidating the biofilm-forming ability and evaluating the structural organization of biofilms and the susceptibility to antimicrobials are crucial for the development of treatment regimens.
